# Novosorb Biodegradable Temporizing Matrix for Reconstruction of Complex Upper-Extremity Wounds

**DOI:** 10.1016/j.jhsg.2024.05.006

**Published:** 2024-07-02

**Authors:** Christopher Jou, Kyle J. Chepla

**Affiliations:** ∗ Department of Plastic Surgery, Cleveland Clinic, Cleveland, OH; † Division of Plastic Surgery, MetroHealth Hospital, Cleveland, OH

**Keywords:** Biodegradable temporizing matrix, Dermal substitute, Novosorb, Reconstruction, Wound healing

## Abstract

**Purpose:**

Dermal matrices can be used in management of upper-extremity wounds to create vascularized wound beds in the setting of exposed bone or tendon. Early outcomes of Novosorb biodegradable temporizing matrix (BTM) demonstrated success when used in the treatment of complex wounds. We hypothesize that BTM is effective for reconstruction of upper-extremity wounds.

**Methods:**

A retrospective review was performed for patients who underwent reconstruction of upper-extremity wounds with BTM between January 2017 and May 2022.

**Results:**

In total, 51 patients (39 males and 12 females) were included. Wound etiology included trauma (n = 30), burn (n = 12), infection (n = 8), and vasopressor-related injury (n = 1). The average size of BTM was 162.5 cm^2^, and the average time from BTM application to wound closure was 90.1 days. Twenty-seven (52.9%) patients required skin grafting, whereas 20 (39.2%) did not and re-epithelialized spontaneously. Those who did not require skin grafting had significantly smaller wound sizes compared to those who required skin grafting (58.5 cm^2^ vs 248.6 cm^2^; *P* = .002). Complications occurred in 14 patients, including infection (n = 5), fluid collection (n = 5), and template dehiscence (n = 4). Wound closure was successful in 92% of patients.

**Conclusions:**

Novosorb BTM is effective for the management of upper-extremity wounds with exposed bone and tendon.

**Clinical relevance:**

In the management of complex upper-extremity wounds with exposed bone and tendon, even when devoid of paratenon or periosteum, Novosorb BTM provides a safe and effective alternative to more complex reconstructive options.

Reconstruction of complex upper-extremity soft tissue wounds with exposed bone and tendon is challenging, and selection of reconstructive technique is guided by the reconstructive ladder. Although these injuries often require local, regional, or free flap reconstruction, skin substitutes and dermal matrices can be used to create a vascularized wound bed amenable to staged reconstruction with skin grafting in wounds with exposed tendon and bone devoid of paratenon and periosteum, respectively.^1^, ^2^, ^3^, ^4^

Skin substitutes can eliminate donor site morbidity associated with flap reconstruction and offer several advantages, including neodermis formation, improved functional and aesthetic outcomes, and decreased scar contracture compared to skin grafting alone.^5^^,^^6^ Among the available dermal matrices, Integra (Integra LifeSciences) is the most widely used biologically derived template and has demonstrated excellent results, particularly in burn reconstruction.^7^, ^8^, ^9^, ^10^, ^11^, ^12^, ^13^, ^14^, ^15^ However, its infection rate has been reported to be as high as 17.9%, and if infection occurs, further surgical revision and reconstruction can be required.^12^^,^^16^

Alternatively, Novosorb biodegradable temporizing matrix (BTM; PolyNovo Limited) is a completely synthetic template, and numerous case reports have highlighted the diverse range of applications for BTM, including upper-extremity reconstruction.^5^^,^^6^^,^^17^, ^18^, ^19^, ^20^, ^21^ Studies comparing BTM to Integra have found similar rates of wound healing with lower rates of skin graft loss and need for secondary surgery.^22^^,^^23^ Furthermore, like Integra, BTM typically requires a second stage skin graft; however, there have been reported cases where skin grafting was not required, and wounds were allowed to heal by re-epithelialization from the wound margins.^17^^,^^22^^,^^23^ In this study, we review our experience and report our outcomes with BTM for reconstruction of complex upper-extremity wounds.

## Materials and Methods

This was a single institution, retrospective institutional review board-approved study of all patients who underwent reconstruction of upper-extremity soft tissue defects with BTM between January 1, 2017, and May 31, 2022. Patients were identified using a current procedure terminology query of our electronic medical records. Patients less than 18 years old, patients with multiple skin substitutes used, patients with wounds that were not located in the upper extremity, and patients lost to follow-up were excluded from our analysis. Demographic data, mechanism of injury, wound size, operative details, need for secondary surgery, and complications were recorded.

### Surgical protocol

At the time of surgery, all nonviable tissue was debrided before the wound was irrigated. Biodegradable temporizing matrix was trimmed to fit, oriented according to the manufacturer’s instructions, and secured either with absorbable sutures or staples. Negative pressure wound therapy or a compressive bolster-type dressing was applied at the discretion of the surgeon. Postoperative antibiotics were given at the discretion of the treating surgeon, and the initial surgical dressing was maintained for 5–7 days before transitioning to a nonadherent dressing with a lightly compressive wrap. Once the BTM was incorporated and the sealing layer removed, patients were given the option of skin grafting or continued local wound care depending on the size of the defect and patient preference for additional surgery.

## Results

**Figure 1:**
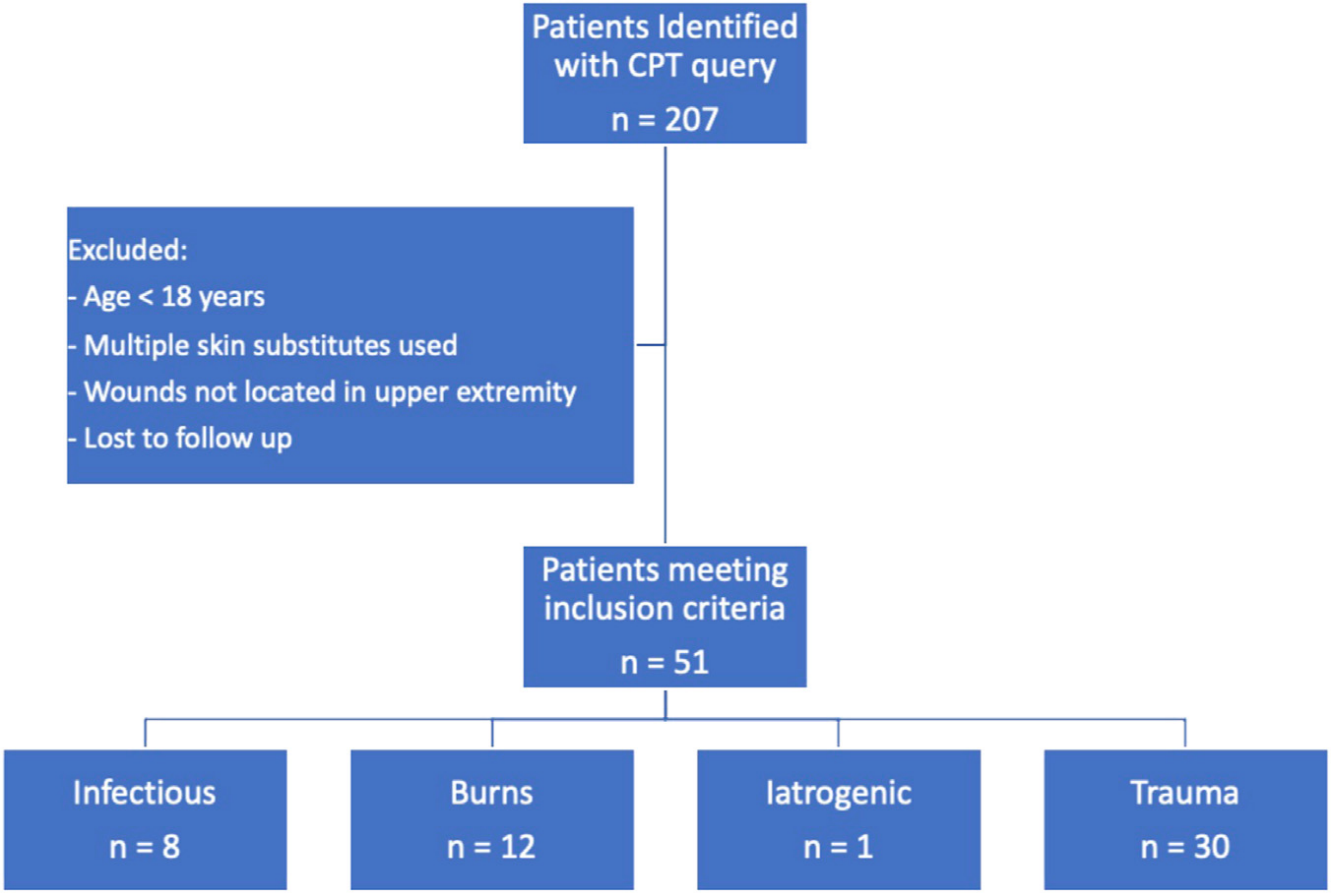
Patient selection flow diagram. CPT, current procedure terminology.

A total of 270 patients were identified using our current procedure terminology query, with a total of 51 patients who met our inclusion criteria (Fig. 1). There were 39 males and 12 females with an average age of 44.3 ± 18.3 years. Nineteen patients (37.3%) were active smokers, and seven patients (13.7%) had diabetes. Wound etiology included trauma (n = 30, 58.8%), burns (n = 12, 23.5%), infection (n = 8, 15.7%), and vasopressor-related injury (n = 1, 2.0%). Twenty-four patients (47.1%) had wounds with exposed bone, and 27 patients (52.9%) had exposed tendon/muscle. The average size of the BTM template used was 162.5 (range 1.5–1,000) cm^2^. The average time from BTM application to complete wound closure was 90.1 (range 11–207) days (Table 1). The mean follow-up was 368.0 ± 262.7 days (range 51–1,216)

**Table 1:** Patient Demographics and Overall Outcomes

Demographics and Outcomes	N	%
Total patients	51	
Gender		
Male	39	76.5%
Female	12	23.5%
Age	44.3 ± 18.3	
Min	13	
Max	97	
Smoker?	19	37.3%
History of diabetes	7	13.7%
Etiology of wound		
Trauma	30	58.8%
Iatrogenic	1	2.0%
Infectious	8	15.7%
Burn	12	23.5%
Depth of wound		
Bone	24	47.1%
Tendon	27	52.9%
Average wound size (cm^2^)	162.5 ± 212.5	
Min	2	
Max	1,000	
Skin graft required?	27	52.9%
Size of skin graft (cm^2^)	166.5 ± 156.8	
Min	10	
Max	600	
Time to skin graft (d)	51.7 ± 22.9	
Min	24	
Max	126	
BTM to wound closure (d)	90.1 ± 51.5	
Min	11	
Max	207	
Follow-up (d)	368.0 ± 262.7	
Min	51	
Max	1,216	

BTM, biodegradable temporizing matrix.

Overall, 47 patients of 51 (92.2%) achieved successful wound closure. Of the four patients who failed reconstruction, two required secondary flap reconstruction with free flap, and two required amputation of the affected finger. Complications occurred in 14 patients and included template infection (n = 5, 9.8%), template fluid collection (n = 5, 9.8%), and template dehiscence (n = 4, 7.9%). Two patients required repeat irrigation and debridement with reapplication of BTM secondary to infection. In the three other patients with infections, BTM was able to be salvaged with incision of the sealing membrane, irrigation, and local wound care.

Twenty patients (39.2%) re-epithelialized spontaneously after removal of the sealing layer and did not require skin grafting. The average wound size for patients without skin grafting was 58.5 (range 2–200) cm^2^, and time to wound closure by secondary intent was 123.7 (range 35–190) days. Twenty-seven patients (52.9%) underwent skin grafting, and the average wound size in this cohort was 248.6 (range 15–1,000) cm^2^. Time to skin grafting from BTM application was 51.7 (range 24–126) days (Table 2). In the 27 patients who required skin grafting, two patients (7.4%) failed skin grafting because of infection (Table 3). One of these patients had partial skin graft loss and was treated with local wound care, whereas the other patient had complete skin graft loss and required repeat skin grafting.

**Table 2:** Comparison Between Skin Grafting and no Skin Grafting

Demographics and Outcome	Skin Grafting	No Skin Grafting	*P* Value
Number	27	20	
Age	39.9 ± 15.4	52.1 ± 21.6	.0143∗
Gender			
Male	20 (74.1%)	15 (75.0%)	.177
Female	7 (25.9%)	5 (25.0%)	.177
Smoker	12 (44.4%)	5 (25.0%)	.043∗
History of diabetes	3 (11.1%)	3 (15.0%)	.153
Etiology of wound			
Trauma	14 (51.9)	14 (70.0%)	.052
Burn	6 (22.2%)	4 (20.0%)	.127
Infectious	7 (25.9%)	2 (10.0%)	.052
Size of wound (cm^2^)	248.6 ± 271.5	58.6 ± 55.4	.002∗
Min	15	2	
Max	1,000	200	
Time to closure (d)	51.7 ± 22.9	123.3 ± 43.9	< .001∗
Complications	6 (22.2%)	6 (30.0%)	.126

∗ *P <* .05.

**Table 3:** Surgical Outcomes and Complications∗

Outcomes and Complications	N	%
Successful wound closure	47	92.1%
Failed wound closure	4	7.9%
Secondary flap because of infection	2	
Amputation†	2	
Patients with complications	14	27.5%
Infection	5	9.8%
Hematoma/seroma	5	9.8%
Partial BTM loss	1	2.0%
Total BTM loss	3	5.9%

∗ BTM, biodegradable temporizing matrix.

† Amputation not related to wounds or wound complication.

When comparing those who required skin grafting and those who were allowed to re-epithelialize secondarily, the group who required skin grafting was on average younger (39.9 ± 15.4 vs 52.1 ± 21.6; *P* = .014) and had more active smokers (44.4% vs 25.0%, *P* = .043). There were no differences in gender, comorbidities, etiology of wounds or complication rates (Table 2). When comparing the size of wounds, those who did not require skin grafting had significantly smaller wounds compared to those who required skin grafting (58.5 cm^2^ vs 248.6 cm^2^; *P* = .002).

## Discussion

Complex upper-extremity wounds present challenges for reconstructive surgeons because of contamination, vascular compromise, and exposure of underlying tendon and bone. Dermal matrices have been used to create a vascularized wound bed, and staged reconstruction with skin grafting serves as an alternative to flap reconstruction for complex wounds. Biodegradable temporizing matrix, a bilayer synthetic matrix, has demonstrated resistance to contamination, reduced scarring, and successful wound closure for burn and traumatic injuries in other studies.^22^^,^^23^

**Figure 2:**
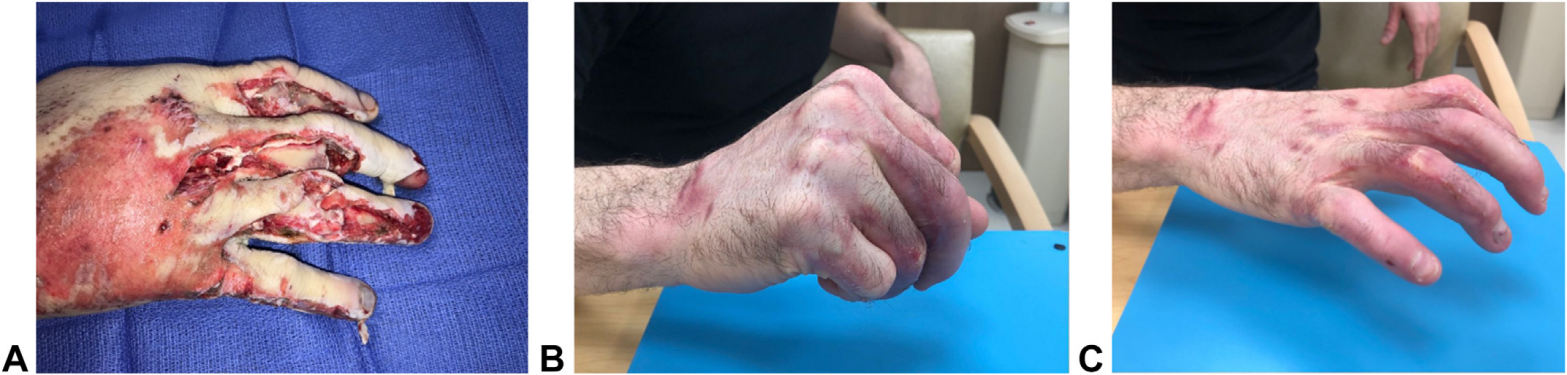
**A** Patient who experienced traumatic injury to right hand resulting in wounds with exposed bone. **B** and **C** After application of BTM, no skin graft was required for complete closure of wound. BTM, biodegradable temporizing matrix.

**Figure 3:**
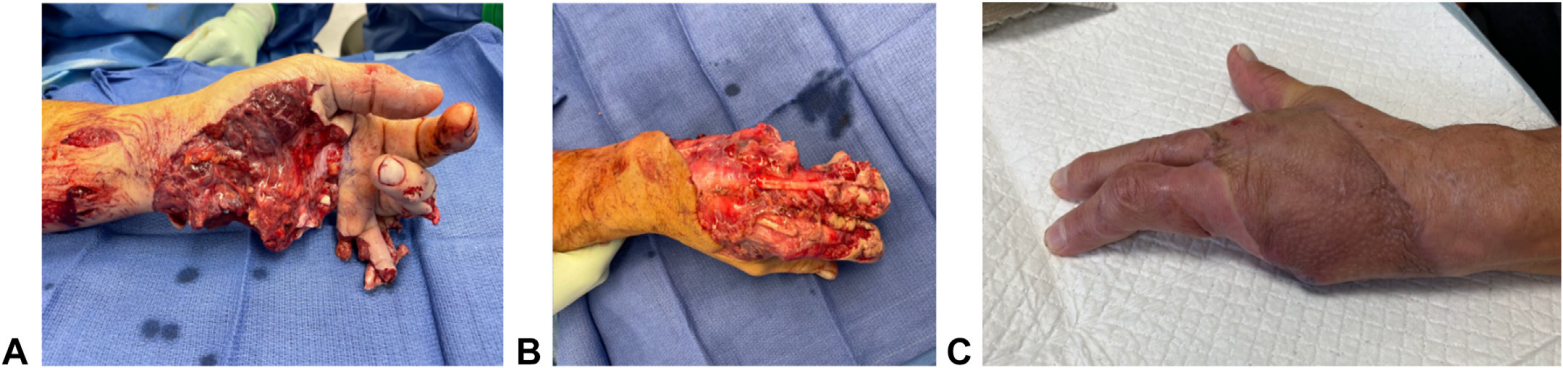
**A** and **B** Patient who experienced partial amputation of left hand after firework injury. **C** After debridement and placement of BTM and complete take of skin graft. BTM, biodegradable temporizing matrix.

In this study, we demonstrated a 92.2% wound closure rate in complex upper-extremity wounds with exposed bone and tendon when BTM was used. Among our cohort, 52.9% of patients required second stage skin grafting, whereas 39.2% re-epithelialized spontaneously. The wounds that healed without skin grafting were smaller when compared to wounds that required skin grafting (Fig. 2). However, wounds as large as 200 cm^2^ were able to heal with local wound care. The average time from BTM placement to second stage skin grafting was 51.7 days, longer than the published 3–4 weeks.^24^^,^^25^ The prolonged time to skin grafting in our cohort was not indicative of delayed vascularization or complications of the BTM template but rather highlights the stability of BTM and ability to graft when convenient for the patient and surgeon.^5^^,^^23^ In polytrauma patients who are unstable or undergoing multiple surgeries, BTM offers the ability to temporize wounds and delay definitive reconstruction until the patients are medically stable (Fig. 3). In wounds that did not require skin grafting, time to wound closure was significantly longer at 150.4 days (range 35–388 days) but eliminated the need for further surgery.

Our study reported a complication rate of 27.5%, similar to findings in other studies.^22^^,^^23^^,^^26^ Despite this, only four patients failed reconstruction with BTM. Among the five patients who had template infections, three were salvaged with local wound care alone and later resulted in successful wound healing. The ability to preserve the template is secondary to the synthetic makeup of BTM, which resists bacterial degradation and contrasts with other biologic derived dermal matrices where infection typically results in loss of dermal template with subsequent need for reapplication or reconstruction using alternate methods.^12^

Like other dermal matrices, BTM creates a vascularized wound bed that is suitable for grafting, and the sealing layer prevents evaporative losses, decreases bacterial contamination, and facilitates dressing changes and wound care. Previous studies that compared BTM to Integra found that BTM had lower rates of skin graft loss and lower need for secondary surgery.^22^ This reduced need for secondary surgery was directly related to the decreased need for staged grafting and is further supported by this study in which almost 40% of were allowed to heal by re-epithelialization. In this study, BTM was also successful for reconstructing defects that previously would have been treated with regional or free flaps. In addition to the decreased need for secondary surgery, this likely represents further potential cost savings by reducing the need for and duration of hospitalization.

There are several limitations to the current study including the retrospective design. Although it was performed at a single institution with a patient population that might vary from other institutions, it has been used by all five plastic and reconstructive surgeons at our hospital for various indications. Further studies evaluating cost-effectiveness, reduced patient morbidity, and functional outcomes are required to better refine indications and expected outcomes when using BTM.

In conclusion, Novosorb BTM is safe and effective for management of complex upper-extremity wounds with exposed bone and tendon even when devoid of paratenon and periosteum as an alternative to flap reconstruction in select patients. Skin grafting may not be necessary in patients with smaller wounds.

## Conflicts of Interest

Dr Chepla is a paid consultant for Polynovo, the manufacturer of Novosorb biodegradable temporizing matrix. No benefits in any form has been received or will be received by the other author related directly to this article.
